# Application of Composite Spectrum in Agricultural Machines

**DOI:** 10.3390/s20195519

**Published:** 2020-09-26

**Authors:** Fernando Feijoo, Francisco Javier Gomez-Gil, Jaime Gomez-Gil

**Affiliations:** 1 Department of Electromechanical Engineering, University of Burgos, 09006 Burgos, Spain; ffeijoo@ubu.es (F.F.); fjggil@ubu.es (F.J.G.-G.); 2 Department of Signal Theory, Communications and Telematics Engineering, University of Valladolid, 47011 Valladolid, Spain

**Keywords:** composite spectrum, monitoring, vibrations, coherent, non-coherent, poly-coherent, predictive maintenance, agricultural machine, combine harvester

## Abstract

Composite spectrum (CS) is a data-fusion technique that reduces the number of spectra to be analyzed, simplifying the analysis process for machine monitoring and fault detection. In this work, vibration signals from five components of a combine harvester (thresher, chopper, straw walkers, sieve box, and engine) are obtained by placing four accelerometers along the combine-harvester chassis in non-optimal locations. Four individual spectra (one from each accelerometer) and three CS (non-coherent, coherent and poly-coherent spectra) from 18 cases are analyzed. The different cases result from the combination of three working conditions of the components—deactivated (off), balanced (healthy), and unbalanced (faulty)—and two speeds—idle and maximum revolutions per minute (RPM). The results showed that (i) the peaks can be identified in the four individual spectra that correspond to the rotational speeds of the five components in the analysis; (ii) the three formulations of the CS retain the relevant information from the individual spectra, thereby reducing the number of spectra required for monitoring and detecting rotating unbalances within a combine harvester; and, (iii) data noise reduction is observed in coherent and poly-coherent CS with respect to the non-coherent CS and the individual spectra. This study demonstrates that the rotating unbalances of various components within agricultural machines, can be detected with a reduced number of accelerometers located in non-optimal positions, and that it is feasible to simplify the monitoring with CS. Overall, the coherent CS may be the best composite spectra formulation in order to monitor and detect rotating unbalances in agricultural machines.

## 1. Introduction

Modern agriculture relies on the intensive use of machines [1]. As mechanization increases, reliance on human operators for the detection of malfunctions is more difficult [2]. Agricultural machines, such as combine harvesters, are complex machines with multiple shafts rotating at different speeds, connected to the engine through pulleys and belts, and supported by bearings [3]. Sensors, microprocessors, and communication devices in modern agricultural machines are used to monitor several operating parameters [4] and enable the possibility of early fault detection in order to reduce breakdowns and down time. Predictive maintenance is based on the surveillance of the machines during their operation, in order to detect the faults prior to breakage [5]. In this way, interventions can be scheduled to prevent failure during the operation of the machine, thus reducing the maintenance costs [6].

Vibration analysis is considered as the most effective and widely used technique for fault detection in the predictive maintenance of machines in industrial applications [5,6,7,8,9]. Frequencies related to the turning speed of the components can be easily identified in the spectra, due to the residual unbalances. Also, damage and wear in any of the shafts can increase the unbalance and the amplitude of the vibration. Therefore, unbalance can be detected as the rotational speed of the shaft increases, because of incremental vibration [10]. 

In complex systems, such as agricultural machines, multiple accelerometers are required for collecting vibration data from all the components to be monitored [11]. Optimum accelerometer positions are the supports of the axes of the rotating components and the direction perpendicular to the rotation axis [10]. Nevertheless, as the vibration propagates along the machine structure with moderate attenuation, the detection of the unbalance is also possible at a location no longer near the vibration source. However, the vibration signal will be more affected by noise, structural resonances, vibrations from the other moving parts and, if driving the machine on uneven soil, induced vibrations. Consequently, measured signal intensity depends on the path from the point of origin of the vibration to the location of each accelerometer. Studies performed in the field of vibration signals applied to agricultural machinery are mainly focused on the vibrations that affect the body of a driver [12,13,14,15,16,17], and, to the best of our knowledge, there are few works on vibration data processing for predictive maintenance [18,19,20].

Vibration data processing for predictive maintenance is a field undergoing continuous advances. Signal analysis in the time domain, the frequency domain, and the time-frequency domain can be used to detect faulty conditions [21]. Fault diagnosis can be performed with these techniques, based on pattern recognition, but they still rely on trained and expert personnel to perform this analysis. Different techniques based on data-mining or artificial intelligence methods have been studied, in order to automatize the diagnosis and to avoid the need for a skilled technician [22,23]. Some of these fault diagnosis techniques, such as support vector machine (SVM) [24] and artificial neural network (ANN) [25], are limited by their feature extraction from the vibration signal analysis [26]. Deep learning (DL) can surmount this limitation by extracting features from raw data [27]. Guo [28] integrated a time-frequency domain technique with a convolutional neural network for diagnosis. Tian [29] used a deep convolutional neuronal network for feature extraction in combination with an immunity antibody algorithm for classification.

In the field of predictive maintenance applied to agricultural machinery, our team has analyzed vibration signals for the detection of the working status of a combine harvester. Ruiz-González et al. [18] applied the SVM-based classifier for estimating the state of various rotating components with a vibration signal acquired from a single point on the machine chassis. And Martínez-Martínez et al. [19], using a single vibration signal, applied an artificial neural network for detecting the status of several rotary components.

The aforementioned artificial intelligence techniques, such as SVM, ANN, and DL, applied to vibration, require extensive vibration-data processing [22]. When the number of components to monitor or the accuracy or the monitorization increases, a higher number of accelerometer signals have to be analyzed for fault detection, which increases the complexity of the data-processing that is required.

Composite spectrum (CS) is a novel procedure that can be used to reduce the number of vibration signals to be processed with artificial intelligence techniques. CS fuses several spectra in one single spectrum maintaining the information of each of them [30,31]. The main advantage of the CS fusion technique is the reduction of the number of spectrums to be analyzed, simplifying the analysis process for condition monitoring of machines. Another advantage of this technique is the reduction of signal noise. Poly-coherent CS has been proposed as an improvement for fault diagnosis in rotating machines, because it retains the vibration phase information [32]. Previous studies using CS have been performed on a test rig to detect shaft misalignment, seal rubbing, and cracks [30,31,32]. In all these studies, the accelerometers were placed directly on the supports of the axes of the rotating components and perpendicular to the rotating axis, which are considered the optimal locations. To our knowledge, only one study using CS technique has been performed on a real machine—a steam turbine generator—for detecting misalignment [33]. CS has been used in conjunction with PCA [34] and ANN [35] for classifying rotating machine faults.

The first objective of this work is to demonstrate the possibility of monitoring and detecting rotating unbalances within various components in agricultural machines with a reduced number of accelerometers, which moreover are not placed in the optimum locations and orientations. The second objective is to study the feasibility of CS fusion technique for this detection, comparing the individual spectra with the non-coherent, coherent and poly-coherent CS. The third objective is to compare the noise related data in all the spectra that are analyzed.

For this purpose, four accelerometers were installed along the chassis of a combine harvester. Access to the optimum points or the optimum direction for the installation of accelerometers within agricultural machines is never straightforward. The accelerometers were not directly installed on the bearing supports but rather was installed at quite some distance from the rotating axis in order to obtain a more general application for different machines with different axis orientations. Furthermore, in three of the four accelerometers, the direction of the accelerometers was not perpendicular to the unbalanced shaft. Five components of a combine harvester are chosen as the most relevant and representatives of the operation of the combine harvester: the thresher, the chopper, the straw walkers, the sieve box, and the engine. These components will be studied in different working conditions: (i) with the thresher and/or the chopper deactivated (off), activated and balanced, and activated and unbalanced; and (ii) at idle and at maximum rotating speed. The combination of these working conditions will give 18 different cases. For each case, four individual spectra and three different formulations of the CS will be studied: the non-coherent, the coherent, and the poly-coherent CS.

## 2. Materials and Methods

This section will be divided into five parts. In Section 1, the equipment used for the data acquisition will be described. In Section 2, the 18 cases under analysis will be summarized, detailing the different working conditions and speed of the five components of the combine harvester. In Section 3, the procedure followed for monitoring the vibration data will be outlined. Explanations may be found in Section 4 on the procedure followed to calculate the non-coherent, coherent, and poly-coherent CS. Finally, the statistical analysis will be reported in the fifth part.

### 2.1. Equipment

A 12-year-old New Holland TC-56 combine harvester (New Holland Agriculture, New Holland, PA, USA) with around 4000 working hours, equipped with a Moresil 718 sunflower header (Moresil SL, Posadas, Spain) with seven rows, was used as the agricultural machine for the tests.

**Figure 1:**
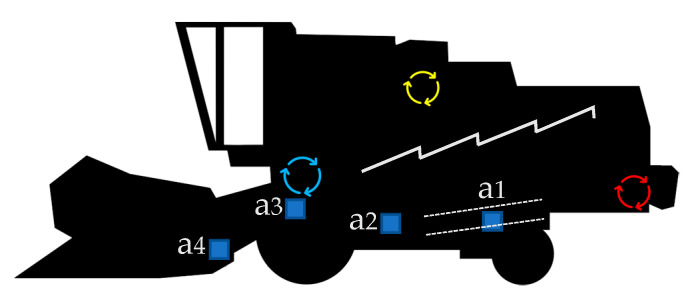
Diagram of a combine harvester. The blue arrows represent the location of the thresher, the yellow ones the location of the engine, and the red ones the location of the chopper. The jagged line represents the straw walkers and the dotted lines, the sieve box. The blue square symbols show the location of the accelerometers, labeled a1–4.

Four PCB 333B40 accelerometers (PCB Group Inc., Depew, NY, USA) were installed on the combine harvester, at arbitrary positions, for recording the vibration signal. Accelerometers a1, a2, and a3 were located on the left side of the chassis, oriented in the lateral direction, at a distance from the rear wheel axle of 300, 1980, and 3006 mm, respectively, and at a height from the floor of 1010, 1040, and 1560 mm, respectively. The fourth accelerometer (a4) was located on the left side of the lower rear beam of the header, at a distance of 1520 mm from the header center, oriented in the longitudinal direction. Accelerometers were positioned by using adhesive wax, permitting accurate measurements within this frequency range. The components and position of the accelerometers are represented in Figure 1.

Vibration signal data were acquired from the accelerometers, using a National Instruments NI-9234 data-acquisition module (National Instruments Corp., Austin, TX, USA) for analog input, mounted on a National Instruments NI CompactDAQ-9172 chassis, connected via USB cable to a laptop computer.

The software used for the data acquisition was NI Sound and Vibration Assistant, and the program used for the data analysis was MATLAB (The MathWorks Inc., Natick, MA, USA).

### 2.2. Case Analyses by the Different Working Conditions and Speed of the Components of the Combine Harvester

The cases under analysis were selected with a combination of different working conditions and speeds of the components of the combine harvester: (i) velocity of the engine and all the other components varied between idle and maximum revolutions per minute (RPM); (ii) the thresher and the chopper varied between off (deactivated) status, balanced (on and healthy) status, and unbalanced (on and faulty) status; and (iii) the straw walkers and the sieve box varied between activated (on) and deactivated (off) status in conjunction with the thresher. The engine was always activated (on). The header of the combine harvester was always deactivated. The combination of the thresher and chopper working conditions and the speed yielded 18 different study cases (D1–D18) shown in Table 1.

### 2.3. Procedure of Vibration Data Acquisition 

A total of 18 different data-acquisition processes were performed, to acquire data in all the status cases listed in Table 2. In all cases, the combine harvester was stationary and with the header deactivated. Unbalances in the chopper are typically caused by blade breakage against stones in the straw, an effect generated in this study by removing one of the chopper blades. Unbalances in the thresher are usually caused when its bars suffer from non-uniform wear due to usage. This effect was generated by attaching an eccentric weight to the thresher.

For each combine harvester status, five 12-second-long measurements were recorded from each of the four accelerometers, at a sample rate of 1706.48 Hz.

Non-coherent, coherent, and poly-coherent CS from the 18 cases were calculated, by dividing each measurement into 11 segments, with 6820 samples each segment, and applying a Hanning window with an overlap of 80%.

The rotation speeds of the different components of the combine harvester (Table 2) were measured with a tachometer, in order to identify the peak relative to the fundamental frequency of each component in the spectra. The speed of the thresher, chopper, straw walkers, sieve box, and engine at idle and maximum RPM are shown in Table 2.

### 2.4. Composite Spectrum Calculation

The calculation of the three different formulations of the CS has been obtained as follow. 

#### 2.4.1. Power Spectrum Density and Cross Power Spectrum Density

The power spectrum density is calculated as follows,

(1) $$S_{xx}\left(f_{k}\right)=\frac{\sum_{r=1}^{n}X^{r}\left(f_{k}\right)\cdot X^{r}{}^{*}\left(f_{k}\right)}{n},\;k=1,2,\;3,\;\ldots ,\;N\;$$

where $S_{xx}\left(f_{k}\right)$ is the power spectrum density, $X^{r}\left(f_{k}\right)$ and $X^{r^{*}}\left(f_{k}\right)$ are the Fourier transform signal and its conjugated series for the *N* frequencies *f_k_* and for the *r*-th fragment of a signal divided in n samples.

The cross power spectrum density is calculated as follows,

(2) $$S_{x_{p}x_{p+1}}\left(f_{k}\right)=\frac{\sum_{r=1}^{n}X_{p}^{r}\left(f_{k}\right)\cdot X_{p+1}^{r}{}^{*}\left(f_{k}\right)}{n},\;k=1,2,\;3,\;\ldots ,\;N$$

where, $X_{p}^{r}\left(f_{k}\right)$ is the Fourier transform for sensor *p*; and $X_{p+1}^{r}{}^{*}\left(f_{k}\right)$ is the conjugated Fourier transform for sensor *p* + 1.

#### 2.4.2. Non-Coherent CS

$S_{CS}\left(f_{k}\right)$ is the non-coherent *CS*, resulting from combining the signals from several accelerometers, calculated as proposed by Elbhbah and Sihna [30,31],

(3) $$S_{CS}\left(f_{k}\right)=\frac{\sum_{r=1}^{n}X_{CS}^{r}\left(f_{k}\right)X_{CS}^{r}{}^{*}\left(f_{k}\right)}{n},\;k=1,2,\;3,\;\ldots ,\;N$$

$\;X_{CS}{}^{r}\left(f_{k}\right)$ and $X_{CS}{}^{r^{*}}\left(f_{k}\right)$ are the composite Fourier transforms and the Fourier transform conjugate for the *r*-th fragment of a signal.

The composite Fourier transform, for *b* accelerometers, is calculated as the geometric means of the cross-power spectrum densities as follows:

(4) $$X_{CS}^{r}\left(f_{k}\right)=\sqrt{{\left(S_{x_{1}x_{2}}^{r}\left(f_{k}\right)\cdot S_{x_{2}x_{3}}^{r}\left(f_{k}\right)\cdot \ldots \cdot S_{x_{\left(b-1\right)}x_{b}}^{r}\left(f_{k}\right)\right)}^{1/\left(b-1\right)}},\;k=1,2,\;3,\;\ldots ,\;N$$

#### 2.4.3. Coherent Cross Power Spectrum Density, Coherent Composite Fourier Transform and Coherent CS

Coherent *CS* has been proposed [30,31], in order to cancel out the contribution of contamination by non-correlated signals from different sources. The coherence function is used to eliminate the contribution of these signals.

(5) $$\gamma_{p\left(p+1\right)}^{2}\left(f_{k}\right)=\frac{{\left|\sum_{r=1}^{n}S_{x_{p}x_{p+1}}^{r}\left(f_{k}\right)\right|}^{2}}{\sum_{r=1}^{n}S_{x_{p}x_{p}}^{r}\left(f_{k}\right)\sum_{r=1}^{n}S_{x_{p+1}x_{p+1}}^{r}\left(f_{k}\right)},\;k=1,2,\;3,\;\ldots ,\;N$$

$S_{x_{p}\gamma_{p\left(p+1\right)}x_{p+1}}^{r}\left(f_{k}\right)$ is the coherent cross power spectrum density for accelerometer signals *p* and *p* + 1, which is calculated as follows:

(6) $$S_{x_{p}\gamma_{p\left(p+1\right)}x_{p+1}}^{r}\left(f_{k}\right)=[X_{p}^{r}\left(f_{k}\right)\cdot \gamma_{p\left(p+1\right)}^{2}\left(f_{k}\right)\cdot X_{p+1}^{r}{}^{*}\left(f_{k}\right)],\;\;k=1,2,\;3,\;\ldots ,\;N$$

The coherent composite Fourier transform for frequency, *f_k_*, is:

(7) $$X_{CCS}^{r}\left(f_{k}\right)=\sqrt{{\left(S_{x_{1}\gamma_{12}x_{2}}^{r}\left(f_{k}\right)\cdot S_{x_{2}\gamma_{23}x_{3}}^{r}\left(f_{k}\right)\cdot \ldots \cdot S_{x_{\left(b-1\right)}\gamma_{\left(b-1\right)b}x_{b}}^{r}\left(f_{k}\right)\right)}^{1/\left(b-1\right)}},\;k=1,2,\;3,\;\ldots ,\;N$$

The coherent *CS* can be calculated as

(8) $$S_{CCS}\left(f_{k}\right)=\frac{\sum_{r=1}^{n}X_{CCS}^{r}\left(f_{k}\right)X_{CCS}^{r}{}^{*}\left(f_{k}\right)}{n},\;k=1,2,\;3,\;\ldots ,\;N$$

$X_{CCS}{}^{r}\left(f_{k}\right)$ and $X_{CCS}{}^{r^{*}}\left(f_{k}\right)$ are the coherent composite Fourier transforms and the conjugated transforms for the r fragment of a signal.

#### 2.4.4. Poly-Coherent CS

Non-coherent and Coherent CS lose phase-angle information as result of the multiplication of composite coherent Fourier Transform by its conjugate series. Yunusa-Kaltungo et al. [32] proposed a poly-coherent CS, $S_{pCCS}\left(f_{k}\right)\;$, to retain the phase-angle information, calculated as follows:

(9) $$S_{CS}\left(f_{k}\right)=\frac{{\left(\sum_{r=1}^{n}X_{1}^{r}\left(f_{k}\right)\cdot \gamma_{12}\cdot X_{2}^{r}\left(f_{k}\right)\cdot \gamma_{23}\cdot X_{3}^{r}\left(f_{k}\right)\cdot \ldots \cdot X_{\left(b-1\right)}^{r}\left(f_{k}\right)\cdot \gamma_{\left(b-1\right)b}\cdot X_{b}^{r}{}^{*}\left(f_{k}\right)\right)}^{\frac{1}{b}}}{n},\;\;k=1,2,\;3,\;\ldots ,\;N$$

### 2.5. Statistical Analysis

All statistical analyses were conducted using Excel statistical functions. The 18 cases were grouped by working conditions (off, balanced, unbalanced) and speed (idle or max RPM). Between-group differences for selected continuous variables (peak amplitudes of the fundamental frequencies) were examined with the Student’s t test for paired samples. The significance level was set at *p* < 0.0001.

Effect sizes are used to describe the strength of a phenomenon, to determine whether differences are of a relevant magnitude. We reported effect size measured by Cohen’s d and effect size (*r*) [36]. Cohen reported the following intervals for d: 0.2 to 0.4: small effect; 0.5 to 0.7: intermediate effect; 0.8 and higher: strong effect. We chose the following labels, in order to classify the magnitude of the differences obtained between groups: A: higher differentiation, if Cohen’s d was >3.5; B: medium differentiation, if Cohen’s d was <3.5); and, C: lower differentiation, if Cohen’s d was <2.5.

The area under the curve (AUC) was used to compare the noise in the different spectra. The AUC was calculated in two ways: (i) for all points integrating the spectrum points from 0 Hz to 120 Hz; and, (ii) for the 50% of the points of lower height. With these two values, an AUC ratio is calculated: AUC of half of the spectrum points that have a value/AUC that is lower than the total integration of the spectrum.

## 3. Results

The comparison between the component peak amplitudes of the rotational speed frequency within the spectra and the noise-related data was analyzed in two ways. In Section 3.1, the spectra of two cases (Cases D14 and D18) will be compared. These cases are shown, in order to provide an overview of the five components in the spectra under analysis. Cases D14 and D18 were choosen as the most representative of the real operation of the harvester, because in both cases all the components under study were activated and working at maximum RPM, with all the components balanced in Case 14 and unbalanced in Case 18. Moreover, the coherence functions are also presented for Case 18. 

In Section 3.2, all cases (D1 to D18) were analyzed and the data were statistically compared.

### 3.1. Analysis and Comparisons of Two Cases, with the Thresher and the Chopper, Both in Balanced (Case D14) and Unbalanced (Case D18) Conditions, and with the Motor at Maximum Rotational Speed

In Section 3.1.1 and Section 3.1.2, the spectra from two of the 18 possible cases presented in Table 1 (cases D14 and D18) are presented. Section 3.1.1 presents the individual spectra from the four accelerometers, and Section 3.1.2 presents the three different formulations of the CS: non-coherent-CS, coherent-CS, and poly-coherent-CS. Section 3.1.3 presents the Coherence functions for case D18, which is the case with the highest noise present in the signal.

#### 3.1.1. Individual Spectra: Identification of the Components by Their Peaks and Comparisons of the Amplitudes of the Peaks in the Four Spectra in Cases D14 and D18

**Figure 2:**
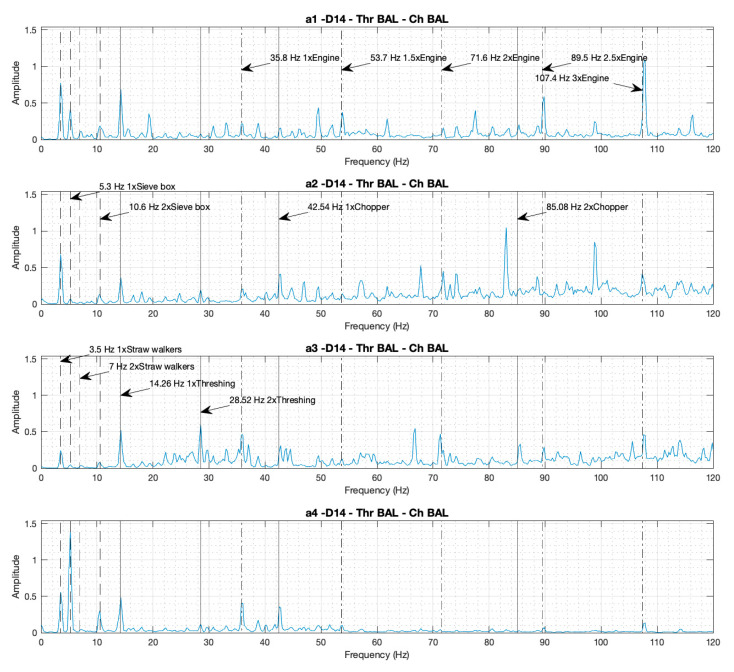
Individual spectra from the four accelerometers (a1–4), with the thresher and the chopper both engaged and balanced, with the motor at maximum rotational speed (Case D14).

**Figure 3:**
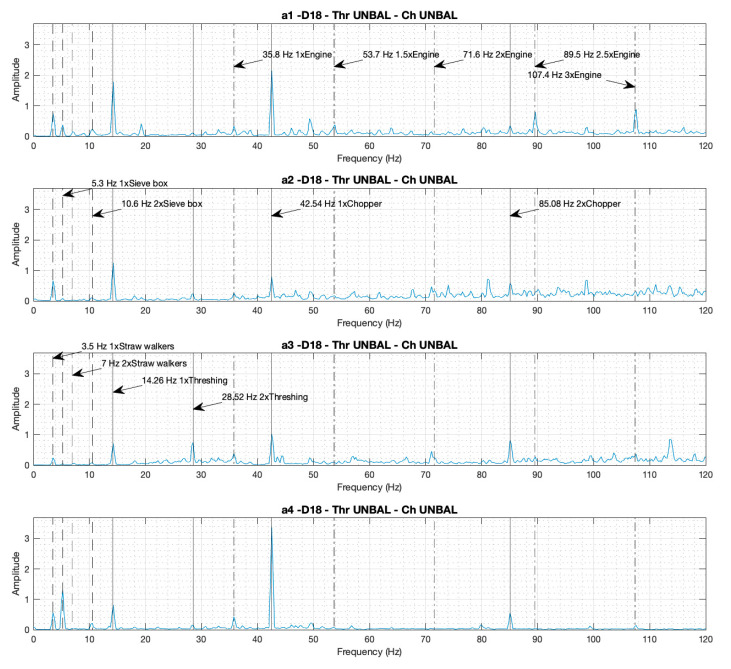
Individual spectra from the four accelerometers (a1–4), with the thresher and the chopper both engaged and unbalanced, with the motor at maximum rotational speed (Case D18).

Figure 2 and Figure 3 presents the individual spectra from the four accelerometers corresponding to cases D14 and D18, respectively. In both cases the combine harvester is working at maximum rotational speed with both the thresher and the chopper engaged, but in case D14 it is working in a balanced (healthy) condition, and in case D18 it is working in an unbalanced (faulty) condition.

In the four individual spectra, the peaks corresponding to the rotational speed of the different components under analysis can be identified. Various peaks are shown at the fundamental frequency, which is the rotational speed: the straw walkers at 3.5 Hz, the sieves box at 5.3 Hz, the thresher at 14.3 Hz, the engine at 35.8 Hz, and the chopper at 42.5 Hz. The peaks corresponding to the harmonic frequencies can also be identified: the straw walkers 2x at 7.0 Hz, the sieve box 2x at 10.6 Hz, the thresher 2x at 28.5 Hz, the chopper 2x at 85.1 Hz, the 1.5x engine frequency at 53.7 Hz, the 2x engine frequency at 71.6 Hz, the 2.5x engine frequency at 89.5 Hz, and the 3x engine frequency at 107.4 Hz.

When we visually compare the amplitudes of the peaks at the fundamental frequencies of the four spectra a0–3 for case D14 (Figure 2, balanced condition), with the a1–4 spectra of case D18 (Figure 3, unbalanced condition), unbalanced (D18) can be shown to imply an increment in the peak heights corresponding to the turning frequency of the components. This increment appears for the thresher 5.4, 4.7, 4.7, and 1.8 times, and for the chopper 10, 2.3, 2.7, and 8.3 times, within spectrums a1–4 respectively. The proportion between the peak increments is not the same in the four spectra due to the dependency on the position and orientation of the accelerometers. The peaks corresponding to the frequencies of the straw walkers, the sieve box, and the engine, imply no increment to the height amplitude when comparing case D18 (Figure 3) with case D14 (Figure 2), because these components remain balanced in all the cases.

#### 3.1.2. Composite Spectra: Identification of the Components by Their Peaks and Comparisons of the Amplitudes of the Peaks in the Non-Coherent, Coherent, and Poly-Coherent CS for Cases D14 and D18

**Figure 4:**
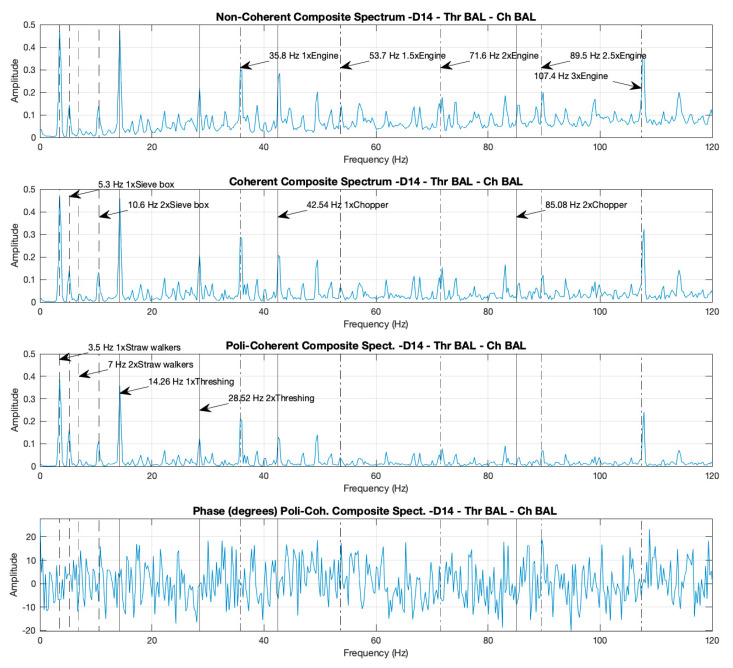
Case D14. Non-coherent, coherent, and poly-coherent composite spectra with the thresher and the chopper both activated and balanced, with the motor at maximum revolutions per minute (RPM).

**Figure 5:**
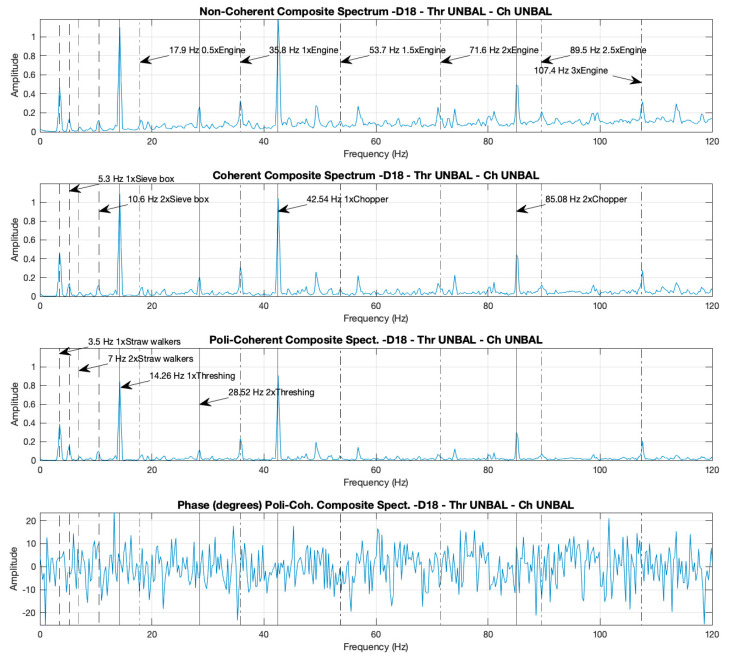
Case D18. Non-coherent, coherent, and poly-coherent composite spectra with the thresher and the chopper both activated and unbalanced, with the motor at maximum RPM.

Figure 4 and Figure 5 present the three different formulations of the CS (non-coherent-CS, coherent-CS, and poly-coherent-CS) corresponding to cases D14 and D18, respectively. CS has been calculated using the previous four spectra presented in Figure 2 and Figure 3, respectively. The non-coherent, coherent, and poly-coherent CS formulations are defined in Equations (4), (8) and (9), respectively.

In the three formulations of the CS for both cases, the peaks corresponding to the rotational speeds of the different components can also be identified: the straw walkers, the sieve box, the thresher drum, the engine, and the chopper, and their harmonic, at the same frequencies as shown previously in the individual spectra. 

When comparing case D18 (Figure 5) with case D14 (Figure 4), an increment can be observed in the peak amplitudes at thresher and chopper frequencies of the non-coherent, coherent, and poly-coherent CS in case D18, due to the unbalance of these components. This increment appears to be for the thresher 2.3, 2.4, and 2.3 times, and for the chopper 4.6, 4.0, and 7.0 times, for the non-coherent, coherent, and poly-coherent CS, respectively. The peaks corresponding to the frequencies of the straw walkers, the sieve box, and the engine, did not increment the height amplitude when case D18 was compared with case D14, because these components remained balanced in all the cases.

#### 3.1.3. Coherence Functions for Case D18 and Noise–Data Comparisons between the Composite and Individual Spectra 

In real machines, individual spectra and non-coherent CS present contamination by non-correlated signals from different sources, referred to as noise. The coherence functions (defined in Equation (5) calculate the correlation between two signals and are used to calculate the coherent and poly-coherent CS, in order to reduce contamination from the non-correlated signals. This technique produces a reduction of the noise in the coherent and poly-coherent CS, with respect to the non-coherent CS, and the individual spectra.

**Figure 6:**
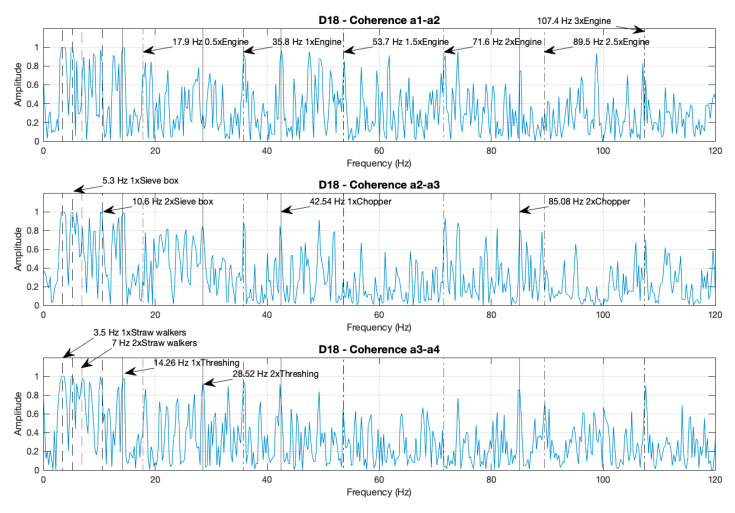
Coherence functions between the combinations of the accelerometers a0–3, placed in the combine harvester, relative to case D18 (the thresher and the chopper both activated and unbalanced, with the motor at maximum RPM).

As an example, Figure 6 presents the three coherence functions between the combinations of the accelerometers placed in the combine harvester, in relation to case D18. It can be appreciated that all the coherence functions reach a value close to 1 for the peaks corresponding to the different components of the machine and its harmonics: straw walkers, sieve box, thresher, chopper, and engine. However, in the areas of the spectrum where there are no components that produce unbalances, the coherence functions are close to zero.

Noise attenuation can be visually appreciated when comparing the spectra. The noise is lower in one spectrum when the number of peaks non-relative to the components are lower and the amplitudes of these peaks reach a value closer to 0. Accordingly, as shown in Figure 2, Figure 3, Figure 4 and Figure 5, the noise is lower in the coherent and poly-coherent CS than in the non-coherent CS and the individual spectra.

### 3.2. Peak Amplitudes Comparison between the Different Spectra in all cases D1-D18

#### 3.2.1. Peak Amplitudes with the Thresher in Deactivation, Activation, and Failure Working Conditions

**Figure 7:**
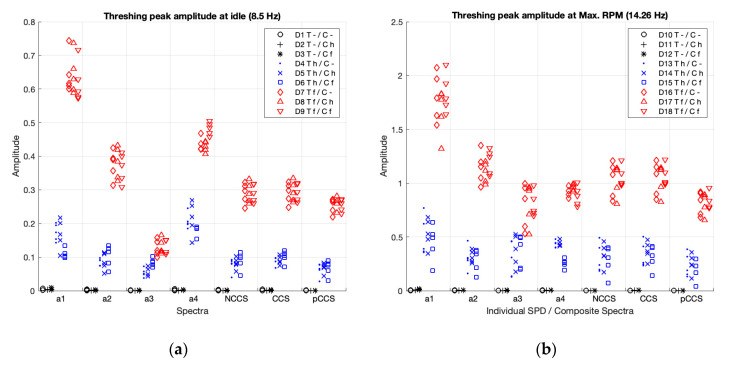
Peak amplitudes for the thresher in the individual spectra (a1–4) and the non-coherent (nCCS), coherent (CCS), and poly-coherent (pCCS) composite spectra, at idle (**a**), and at maximum RPM (**b**). In blue, the balanced cases; in red, the unbalanced cases; and, in black, the cases where the components are deactivated.

Figure 7 and Table 3 present the peak amplitudes comparisons at the fundamental frequency, which are the rotational speed for the thresher, in the individual spectra (a1–4), and in the CS (non-coherent, coherent, and poly-coherent), for the 18 cases in the study (D1–D18). In Figure 7, blue shows the cases with balanced thresher (D4–D6, D13–D15), red shows the cases with an unbalanced thresher (D7–D9, D16–D18), and black shows the cases with the thresher deactivated (D1–D3, D10–D12).

As visually shown in Figure 7, for the thresher, the activation (balanced condition, red cases) and the failure (unbalanced condition, blue cases) can be clearly detected by the accelerometers (a1, a2 and a4), both at idle speed (cases D1 to D9) and at maximum RPM (cases D10 to D18). However, the accelerometer, a3, differentiated between activation and failure modes quite unclearly.

Statistical comparisons in Table 3 show that, for all individual spectra and for the three CS, the non-balanced conditions scored significantly higher than the balanced conditions, and the balanced conditions also scored significantly higher than the deactivated conditions. The magnitude of the differences between all comparisons can be considered as a strength according to Cohen (Cohen’s d > 0.8), i.e., the spectra differentiate the status. Nevertheless, when comparing the effect size of all accelerometers (Table 3), we found, in concordance with previous visual analysis (Figure 7), that the spectra of accelerometer a3 showed the lowest effect size when comparing balanced vs. deactivated conditions (Cohen’s d = 3.2 at max. RPM), and unbalanced vs. balanced conditions (Cohen’s d = 3.3 at idle and 2.9 at max. RPM). And the spectra for the pC-CS also showed a lower effect size when comparing balanced vs. deactivated conditions (Cohen’s d = 3.4 at maximum RPM).

#### 3.2.2. Peak Amplitudes with the Chopper in Deactivation, Activation, and Failure Working Conditions

**Figure 8:**
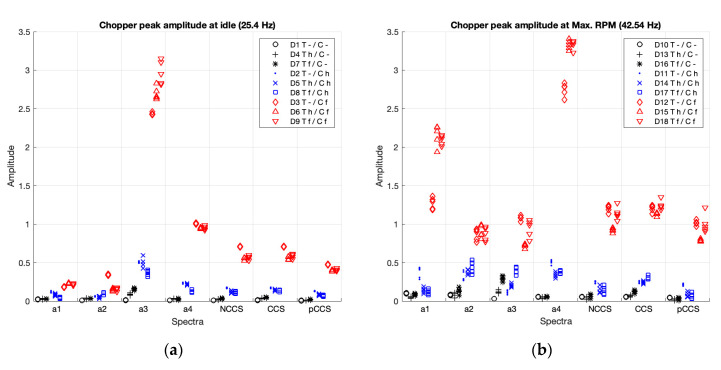
Peak amplitudes of the chopper in the individual spectra (a1–4) and the non-coherent (nCCS), coherent (CCS), and poly-coherent (pCCS) composite spectra at idle (**a**) and at maximum RPM (**b**). The balanced cases are shown in blue, the unbalanced cases in red, and deactivated components, in black.

Figure 8 and Table 4 present the peak amplitude comparisons for the chopper, in the individual spectra (a1–4), and in the composite spectra (non-coherent, coherent, and poly-coherent), for the 18 cases under study (D1–D18). In Figure 8, blue shows the cases with the chopper balanced (D2, D5, D8, D11, D14, D17); red shows the cases with the chopper unbalanced (D3, D6, D9, D12, D15, D18); and black marks the cases with the chopper deactivated (D1, D4, D7, D10, D13, D16).

As visually shown in Figure 8, accelerometers a1 and 2 for the chopper at idle speed (Figure 8a) showed no clear differentiation between activation (blue) vs. off (black), nor between activation (blue) vs. failure (red). And at maximum RPM (Figure 8b), accelerometers a1 and 3, and nCCS and pCCS, showed no clear differentiation between activation (blue) vs. off (black).

Statistical comparisons (Table 4) showed, in accordance with previous visual analysis (Figure 8), that when comparing balanced vs. off status, the effect sizes of the peak differences were lower for the accelerometers a1 and a2 at idle RPM (Cohen’s d = 2.2), and for maximum RPM were lower for the spectra a1 (Cohen’s d = 1.4), a3 (Cohen’s d = 0.8), non-coherent CS (Cohen’s d = 2.6), and poly-coherent CS (Cohen’s d = 1.7). Moreover, when comparing unbalanced vs. balanced status, the effect size was lower for the accelerometer a2 at idle speed (Cohen’s d = 2.1).

#### 3.2.3. Peak Amplitudes with the Straw Walkers and Sieve Box in Deactivation and Activation Working Conditions

**Figure 9:**
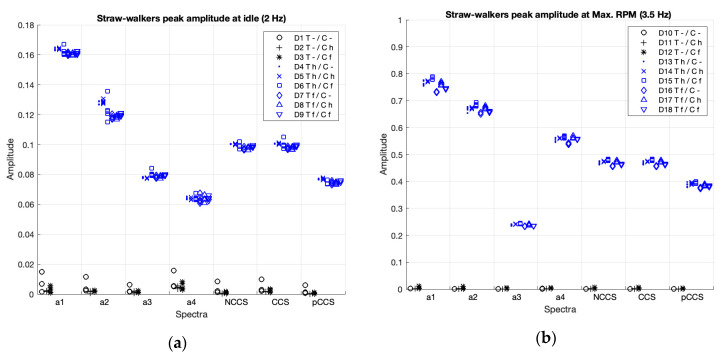
Peak amplitudes of the straw walkers in the individual spectra (a1–4) and the non-coherent (nCCS), coherent (CCS), and poly-coherent (pCCS) composite spectra, at idle (**a**), and at maximum RPM (**b**). The cases where the components are activated are shown in blue and the deactivated components are shown in black.

**Figure 10:**
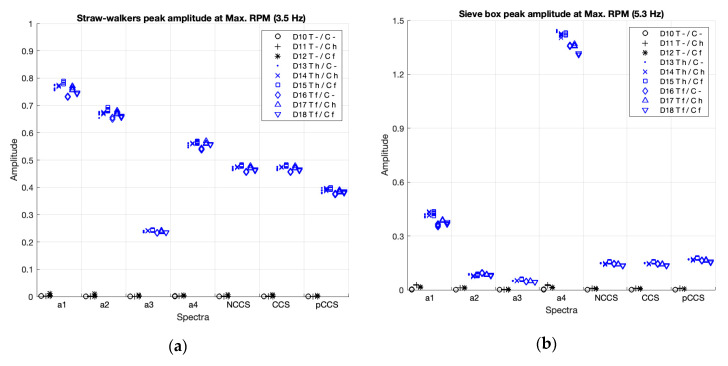
Peak amplitudes of the sieve box in the individual spectra (a1–4) and the non-coherent (nCCS), coherent (CCS), and poly-coherent (pCCS) composite spectra, at idle (**a**), and at maximum RPM (**b**). The balanced cases are shown in blue and the deactivated components are shown in black.

Figure 9 and Table 5 present the peak amplitudes comparisons for the straw walker, and Figure 10 and Table 6 for the sieve box. In this study no unbalanced components were induced. Therefore, in Figure 9 and Figure 10, the cases with both components activated, because the thresher is activated (cases D4–D9, D14–D18), are shown in blue, and the cases with the thresher deactivated (D1–D3, D10–D12) are shown in black.

As can be visually (Figure 9 and Figure 10) and statistically (Table 5 and Table 6) shown, in all the spectra the peak amplitudes clearly differentiate activation (blue) vs. deactivation (black) of the straw walkers and the sieve box, with a very strong effect size. 

#### 3.2.4. Summary of the Magnitude of the Differences of the Peak Amplitudes for Each Spectrum According to the Different Component Status

Table 7 summarizes the magnitude (effect size) of the differences of the peak amplitudes according to the status (deactivated, activated (healthy) or unbalanced (faulty)) of the components (thresher, chopper, straw walkers and sieve box) at idle and at maximum RPM. 

As shown in Table 7, the four individual spectra and the three CS clearly show the differences between the three working conditions (off, balanced, unbalanced) for the four components (Cohen’s d > 0.8). Each spectrum was divided into three categories for comparison by effect size: A: higher differentiation (Cohen’s d > 3.5), B: medium differentiation (Cohen’s d < 3.5), and C: lower differentiation (Cohen’s d < 2.5).

The individual spectra of accelerometers a1 and a2, for the chopper, showed a lower capacity (Cohen’s d < 2.5) for differentiating between balanced vs. off status at both speeds, and unbalanced vs. balanced status at idle speed. Accelerometer a3, for the thresher, showed a medium differentiation (Cohen’s d < 3.5) between balanced vs. off status at Max RPM, and between unbalanced vs. balanced status at both speeds; and, for the chopper, showed a lower capacity (Cohen’s d < 2.5) for differentiating balanced vs. off status at Max RPM. The individual spectra from accelerometer a4 provided a clear differentiation for all the cases (Cohen’s d > 3.5).

The poly-coherent CS provided a medium differentiation (Cohen’s d < 3.5) when comparing balanced vs. off status at Max RPM for the thresher (Cohen’s d < 3.5) and a lower differentiation (Cohen’s d < 2.5) for the chopper. The non-coherent CS provided a medium differentiation (Cohen’s d < 3.5) when comparing balanced vs. off status at Max RPM for the chopper. And the coherent CS provided a clear differentiation for all the cases (Cohen’s d > 3.5).

In summary, the a4 individual spectra and the coherent CS presented the best capacity for differentiating the three working conditions (off, balanced, unbalanced). 

#### 3.2.5. Area Under the Curve Comparisons in the Different Spectra (Individuals, Non-Coherent CS, Coherent CS, and Poly-Coherent CS) in all Cases (D1–D18) 

Table 8 presents the area under the curve (AUC) ratios for all case studies, and the percentage reduction when comparing the ratios of the poly-coherent CS with the ratios of the coherent CS, non-coherent CS and the summation of the four individual spectra.

As shown in Table 8, the poly-coherent CS presented the higher noise reduction, followed in order by the coherent CS, the non-coherent CS, and the individual spectra. The average percentage of the ratio reduction of the poly-coherent CS was 34.9% compared with the coherent CS, 52.2% compared with the non-coherent CS, and 38.8% compared with the summation of the four individual spectra. 

## 4. Discussion

The first relevant result of this study is that rotating unbalances of various components within agricultural machines can be monitored and detected with a reduced number of accelerometers, which are neither directly placed on the bearing supports, nor are they always perpendicular to the rotating axis. As it can be seen in the result section (Figure 2, Figure 3, Figure 4 and Figure 5), the frequencies corresponding to the different components, such as thresher, chopper, straw walkers, sieve box and engine, were identified by the four accelerometers. So, all five components are well identified across all the spectra, however some components are better identified in one spectrum, and other components in another. In some cases, likewise, the peak corresponding to the rotation frequency is better identified in one spectrum, and the harmonic peaks are better identified in another. Additionally, depending on the combination of the three working conditions of the thresher and the chopper (deactivated, activated (healthy) or unbalanced (faulty), at idle or at maximum RPM), the peaks are better identified in one spectrum or in other. In our study, only accelerometer a4, positioned facing in the direction of the vibration in the combine harvester header, gives a clear differentiation for all the cases (Table 7), even though it was the farthest from all the five studied components. The other accelerometers a1–3, which can also detect activation and failure of the components, were not located in the direction of the vibration. Moreover, the capacity to identify the on/off condition of the straw walker and the sieve box is higher (higher effect size) than for the other components; which can be explained by their greater field of movement, as they are located in peripheral positions. In summary, these results suggest that an arbitrary and unusual location of the accelerometers may be useful for monitoring and fault diagnosis for other types of agricultural machines. To our knowledge, no previous studies have been published on monitorization and detection of rotating unbalances of various components within real machines using accelerometers that are not placed on the bearing supports or perpendicular to the rotating axes.

The second important result is that the use of CS, coherent, non-coherent, and poly-coherent for the monitorization and the detection of rotating unbalances in agricultural machines is feasible. CS retains the relevant information from the individual spectra, reducing the number of spectra to be analyzed, and thereby, simplifying the analysis process. Frequencies corresponding to the monitored components can be easily identified in the CS. The three composite spectra formulations retain the relevant information for the monitoring and fault detection of the components (as shown when comparing CS with individual spectra from each accelerometer). In our study, the three different formulations of the CS clearly differentiated all the component statuses of the thresher, the chopper, the straw walkers and the sieve box, except for the activation versus the deactivation status of the chopper and the thresher at maximum RPM, which was not so clear (lower effect size) in the non-coherent CS for the chopper, and poly-coherent CS for the chopper and the thresher. These results suggest that the coherent CS may be the best composite spectra formulation, in order to monitor and to detect rotating unbalances in agricultural machines. Future research is needed, in order to confirm this finding. In summary, this study prompts the thought that monitoring and fault diagnosis for all the machine components can be based on the analysis of a single CS instead of using the individual spectra set from several accelerometers. A review of the literature shows that the few previous studies using the CS technique were performed mainly over a test rig [25,26,27], to monitor shaft misalignment, shaft cracking, and seal rubbing. Only one study was applied to a real machine, a turbo generator, where the misalignment was detected with the fusion of fourteen accelerometers [28]. In contrast, in the present study we used only four accelerometers for monitoring up to five components.

The third result of this study showed that both the coherent and the poly-coherent CS presented an overall noise reduction in comparison with the non-coherent CS (Figure 4 and Figure 5) and with the individual spectra (Figure 2 and Figure 3). This result is in concordance with previous studies performed over a test rig [25,26,27,28]. This noise reduction of the coherent and poly-coherent CS can be important for agricultural machines, because they work a noisy environment and with multiple components that can produce noise due, for instance, to axel turns at several velocities, belts, chains, bars with friction, oscillation movements, etc.

The current study has several strengths. First, it is an innovative study because, as far as we know, this is the only study with arbitrarily positioned accelerometers, not on the supports of the rotating shafts, and at quite some distance from the bearing supports, whereas the accelerometers were placed on the rotating supports in previous studies. Although this arbitrary positioning makes detection and monitoring of rotating unbalances more difficult, the results of this study have proven its feasibility. The second innovation of this study is that, as far as we know, this is the first study that has applied the CS technique for diagnosis of rotating unbalances and the second study applied on a real machine. Finally, as an advance, compared with previous studies, the monitoring system proposed in this study simplified the monitoring process and can be easily implemented in other types of machines, for several reasons: (i) the accelerometers do not have to be located only on the shafts supports, and can be positioned at a distance from the vibration source. This is an important aspect, due to the difficulty of positioning the accelerometers at optimal locations and orientations within certain machines. (ii) Besides, the accelerometers can be located arbitrarily, with no need to know the best place to measure the vibration signal in advance, because the use of the CS retains information on all the individual spectra. (iii) This monitoring system requires a reduced number of accelerometers in comparison with the standard mode of positioning a pair of accelerometers on each rotating shaft. In this way, each accelerometer that is installed detects the vibration signal of all the components of the machine. This is a great advantage in modern agricultural machines, which have multiple shafts rotating at different speeds, and monitoring each individual component would require a high number of accelerometers. And, (iv) using the CS means that there is only one spectrum for analysis, in comparison with the traditional procedures.

The main limitation of the use of CS is that it requires additional post-processing of the signal, in order to obtain the CS, with respect to the analysis of the individual spectra. However, the postprocessing work is compensated by the reduction in the number of spectra to be analyzed. Another limitation is that since the location of the accelerometers is arbitrarily chosen, the best location for the accelerometers will not be known a priori. Finally, other rotating components that generate vibration such as the beater cylinder, the cleaning fan, the hydrostatic pump, the elevator chains, the augers, and the drum variators, were not studied in this work. Some of these components also generate friction-induced vibration. Nevertheless, despite these additional sources of vibrations, the CS was capable of detecting the unbalanced elements under study.

## 5. Conclusions

In summary, this study suggests that (1) it is possible to monitor and to detect the rotating unbalances of various components within agricultural machines with a reduced number of accelerometers, which are moreover not placed in the usual optimum positions and orientations. (2) The use of the CS (coherent, non-coherent, and poly-coherent) for monitoring and detecting rotating unbalances in agricultural machines is feasible. CS retains the relevant information for the fault diagnosis, reducing the number of spectra required. (3) Coherent and poly-coherent CS reduce the data noise with respect to the non-coherent CS and the individual spectra, in noisy environments. Overall, the coherent CS may be the best composite spectra formulation, in order to monitor and to detect rotating unbalances in agricultural machines.

The monitoring system proposed in this study has not only shown its sensitivity to fault detection but has also simplified the monitoring process and can be easily implemented in other types of machines.

## Figures and Tables

**Table 1:** Cases studied according to the combination of the component working conditions status (off, balanced, and unbalanced) and velocity (idle and maximum revolutions per minute (RPM)).

Thresher Status	Chopper Status	Idle	Max. RPM
*Off (Deactivated)*	*Off*	*D1*	*D10*
	*Balanced*	*D2*	*D11*
	*Unbalanced*	*D3*	*D12*
*Balanced (on and Healthy)*	*Off*	*D4*	*D13*
	*Balanced*	*D5*	*D14*
	*Unbalanced*	*D6*	*D15*
*Unbalanced (on and Faulty)*	*Off*	*D7*	*D16*
	*Balanced*	*D8*	*D17*
	*Unbalanced*	*D9*	*D18*

**Table 2:** Speeds of the components of the combine harvester at idle and at maximum RPM.

Regime	Thresher	Chopper	Straw Walkers	Sieve Box	Engine
*Idle*	*8.5 Hz (508.3 RPM)*	*25.4 Hz (1524 RPM)*	*2 Hz (180 RPM)*	*3 Hz (120 RPM)*	*21.4 Hz (1280 RPM)*
*Max. RPM*	*14.26 Hz (839.5 RPM)*	*42.54 Hz (2517 RPM)*	*3.5 Hz (318 RPM)*	*5.3 Hz (210 RPM)*	*35.8 Hz (2144 RPM)*

**Table 3:** Sample description and statistical comparison of the peak amplitudes of the thresher, detected in individual spectra (a1–4), and the non-coherent (nCCS), coherent (CCS), and poly-coherent (pCCS) composite spectra, under different working conditions (deactivated, balanced and non-balanced) and at different speeds (at idle and at max. RPM).

		Deactivated	Balanced	Unbalanced	Balanced vs. Deactivated			Unbalancedb vs. Balanced		
		*N* = 15 M (SD)	*N* = 15 M (SD)	*N* = 15 M (SD)	t	p	d	t	p	d
a1	Idle	0.0043 (0.0021)	0.1502 (0.0413)	0.6340 (0.0562)	9.61	*	5.2	31.23	*	9.8
	Max.	0.0087 (0.0043)	0.4934 (0.1566)	1.7703 (0.2050)	8.73	*	4.4	21.97	*	7.0
a2	Idle	0.0017 (0.0011)	0.0915 (0.0244)	0.3736 (0.0415)	9.90	*	5.4	22.28	*	8.3
	Max.	0.0045 (0.0011)	0.3007 (0.0890)	1.1472 (0.1200)	9.19	*	4.7	25.13	*	8.0
a3	Idle	0.0010 (0.0004)	0.0640 (0.0190)	0.1286 (0.0210)	9.16	*	4.9	9.62	*	**3.2**
	Max.	0.0027 (0.0011)	0.3460 (0.1471)	0.8001 (0.1654)	7.09	*	**3.3**	8.56	*	**2.9**
a4	Idle	0.0022 (0.0013)	0.2008 (0.0342)	0.4479 (0.0305)	13.49	*	8.5	25.76	*	7.6
	Max.	0.0048 (0.0012)	0.3793 (0.0973)	0.9146 (0.0667)	10.18	*	5.4	18.89	*	6.4
nCCS	Idle	0.0009 (0.0007)	0.0822 (0.0209)	0.2907 (0.0272)	10.25	*	5.7	27.75	*	8.6
	Max.	0.0015 (0.0008)	0.3065 (0.1194)	1.0376 (0.1286)	7.58	*	3.6	19.21	*	5.9
CCS	Idle	0.0015 (0.0008)	0.0936 (0.0147)	0.2928 (0.0268)	14.26	*	9.2	23.45	*	9.2
	Max.	0.0036 (0.0011)	0.3377 (0.1012)	1.0467 (0.1247)	9.13	*	4.7	19.84	*	6.2
pCCS	Idle	0.0006 (0.0006)	0.0660 (0.0185)	0.2538 (0.0195)	9.61	*	5.2	35.53	*	9.9
	Max.	0.0008 (0.0006)	0.2308 (0.0969)	0.8178 (0.0898)	7.18	*	**3.4**	20.70	*	6.3

M (SD): median (standard deviation). *N* = number of cases in that condition (deactivated, balanced or non- balanced) x number of trials in each case (five) = 3 × 5. * *p* < 0.0001. Bold font highlights the lower effect sizes.

**Table 4:** Sample description and statistical comparison of the peak amplitudes of the chopper detected in individual spectra (a1–4), and the non-coherent (nCCS), coherent (CCS), and poly-coherent (pCCS) composite spectra, under different working conditions (deactivated, balanced and non-balanced) and at different speeds (at idle and at max. RPM).

		Deactivated	Balanced	Unbalanced	Balanced vs. Deactivated			Unbalance vs. Balanced		
		*N* = 15 M (SD)	*N* = 15 M (SD)	*N* = 15 M (SD)	t	p	d	t	p	d
a1	Idle	0.0257 (0.0050)	0.0788 (0.0344)	0.2082 (0.0207)	5.15	*	**2.2**	12.45	*	4.6
	Max.	0.0780 (0.0239)	0.2083 (0.1272)	1.8377 (0.4242)	3.62	*	**1.4**	11.08	*	5.2
a2	Idle	0.0272 (0.0112)	0.0676 (0.0243)	0.2133 (0.0933)	5.58	*	**2.2**	5.25	*	**2.1**
	Max.	0.0973 (0.0342)	0.3802 (0.0743)	0.8773 (0.0754)	11.94	*	4.9	22.20	*	6.6
a3	Idle	0.0946 (0.0579)	0.4571 (0.0775)	2.6982 (0.2461)	15.33	*	5.4	21.70	*	12.3
	Max.	0.1457 (0.1142)	0.2394 (0.1279)	0.9128 (0.1658)	2.14	*	**0.8**	13.68	*	4.5
a4	Idle	0.0224 (0.0094)	0.1935 (0.0446)	0.9699 (0.0310)	10.68	*	5.5	65.42	*	20.2
	Max.	0.0482 (0.0104)	0.4060 (0.0770)	3.1347 (0.2913)	11.85	*	6.5	21.04	*	12.8
nCCS	Idle	0.0227 (0.0120)	0.1354 (0.0286)	0.6069 (0.0760)	12.19	*	5.3	17.08	*	8.2
	Max.	0.0480 (0.0217)	0.1745 (0.0640)	1.0872 (0.1347)	6.48	*	**2.6**	20.18	*	8.7
CCS	Idle	0.0348 (0.0149)	0.1466 (0.0206)	0.6168 (0.0692)	18.06	*	6.3	17.14	*	9.2
	Max.	0.0807 (0.0325)	0.2631 (0.0351)	1.1912 (0.0669)	17.32	*	5.4	41.06	*	17.4
pCCS	Idle	0.0121 (0.0066)	0.0934 (0.0279)	0.4254 (0.0380)	8.76	*	4.2	31.77	*	10.0
	Max.	0.0283 (0.0164)	0.1184 (0.0740)	0.9342 (0.1289)	4.22	*	**1.7**	20.56	*	7.8

M (SD): median (standard deviation). *N* = number of cases in that condition (deactivated, balanced or non-balanced) x number of trials in each case (five) = 3 × 5. * *p* < 0.0014. Bold font highlights the lower effect sizes.

**Table 5:** Sample description and statistical comparison of the peak amplitudes of the straw walkers in the individual spectra (a1–4) and the non-coherent (nCCS), coherent (CCS), and poly-coherent (pCCS) composite spectra, under two working conditions (deactivated, activated) and (at idle and at max. RPM) speeds.

		Deactivated	Activated	Activated vs. Deactivated		
		*N* = 15 M (SD)	*N* = 45 M (SD)	t	p	d
a1	Idle	0.0037 (0.0038)	0.1620 (0.0019)	47.87	*	47.3
	Max.	0.0030 (0.0024)	0.7594 (0.0178)	735.19	*	73.0
a2	Idle	0.0027 (0.0027)	0.1226 (0.0052)	563.73	*	33.2
	Max.	0.0020 (0.0022)	0.6679 (0.0117)	1391.65	*	96.2
a3	Idle	0.0019 (0.0014)	0.0788 (0.0014)	175.83	*	55.5
	Max.	0.0012 (0.0010)	0.2379 (0.0038)	2547.26	*	102.4
a4	Idle	0.0057 (0.0033)	0.0639 (0.0017)	27.80	*	19.8
	Max.	0.0024 (0.0010)	0.5565 (0.0091)	1049.58	*	105.2
nCCS	Idle	0.0015 (0.0022)	0.0989 (0.0016)	75.13	*	48.3
	Max.	0.0010 (0.0016)	0.4692 (0.0080)	1539.22	*	98.0
CCS	Idle	0.0026 (0.0023)	0.0992 (0.0018)	75.11	*	44.4
	Max.	0.0016 (0.0015)	0.4692 (0.0080)	1443.09	*	98.3
pCCS	Idle	0.0009 (0.0016)	0.0754 (0.0015)	115.25	*	48.1
	data	0.0006 (0.0008)	0.3858 (0.0072)	902.67	*	92.6

M (SD): median (standard deviation). *N* = number of cases in that condition (deactivated or balanced) x number of trials in each case (five). * *p* < 0.0001.

**Table 6:** Sample description and statistical comparison of the peak amplitudes of the sieve box in the individual spectra (a1–4) and the non-coherent (nCCS), coherent (CCS), and poly-coherent (pCCS) composite spectra, under different working conditions (deactivated, activated) and speeds (at idle and at max. RPM).

		Deactivated	Activated	Activated vs. Deactivated		
		*N* = 15 M (SD)	*N* = 45 M (SD)	t	p	d
a1	Idle	0.0101 (0.0106)	0.1216 (0.0406)	20.48	*	4.7
	Max.	0.0156 (0.0113)	0.3962 (0.0269)	351.32	*	21.2
a2	Idle	0.0084 (0.0090)	0.1449 (0.0337)	36.32	*	6.9
	Max.	0.0082 (0.0055)	0.0848 (0.0058)	69.58	*	13.7
a3	Idle	0.0039 (0.0042)	0.1190 (0.0163)	83.57	*	12.1
	Max.	0.0018 (0.0010)	0.0498 (0.0045)	140.03	*	17.8
a4	Idle	0.0056 (0.0043)	0.7263 (0.0395)	222.33	*	33.2
	Max.	0.0139 (0.0106)	1.3857 (0.0460)	669.05	*	49.6
nCCS	Idle	0.0052 (0.0063)	0.1716 (0.0313)	50.11	*	9.4
	Max.	0.0055 (0.0039)	0.1455 (0.0065)	559.51	*	28.6
CCS	Idle	0.0060 (0.0063)	0.1717 (0.0313)	49.53	*	9.3
	Max.	0.0058 (0.0038)	0.1459 (0.0065)	589.59	*	28.8
pCCS	Idle	0.0032 (0.0038)	0.1494 (0.0267)	47.40	*	9.9
	Max.	0.0051 (0.0038)	0.1658 (0.0072)	747.80	*	31.1

M (SD): median (standard deviation). *N* = number of cases in that condition (deactivated or balanced) x number of trials in each case (five). * *p* < 0.0001.

**Table 7:** Magnitudes (effect sizes) of the capacity to differentiate the status (deactivated, activated or unbalanced) of the components of a combine harvester functioning at two speeds (idle and maximum RPM) in the four individual spectra (a1–4) and in the non-coherent (nCCS), coherent (CCS), and poly-coherent (pCCS) composite spectra.

	Thresher				Chopper				Straw Walkers		Sieve Box	
	Balanced vs. Deactivated		Unbalanced vs. Balanced		Balanced vs. Off Status		Unbalanced vs. Balanced		Balanced vs. Deactivated		Balanced vs. Deactivated	
	Idle	Max RPM	Idle	Max RPM.	Idle	Max. RPM	idle	Max RPM	Idle	Max RPM	Idle	Max RPM
a1	A	A	A	A	C	C	A	A	A	A	A	A
a2	A	A	A	A	C	A	C	A	A	A	A	A
a3	A	B	B	B	A	C	A	A	A	A	A	A
a4	A	A	A	A	A	A	A	A	A	A	A	A
nCCS	A	A	A	A	A	B	A	A	A	A	A	A
CCS	A	A	A	A	A	A	A	A	A	A	A	A
pCCS	A	B	A	A	A	C	A	A	A	A	A	A

Abbreviations. Effect sizes are represented by A: higher differentiation (Cohen’s d > 3.5), B: medium differentiation (Cohen’s d < 3.5); C: lower differentiation (Cohen’s d < 2.5).

**Table 8:** Noise related data (AUC ratio) and comparisons of percentage reduction between the s the non-coherent (nCCS), coherent (CCS), and poly-coherent (pCCS) composite spectra, and the summary of the four individual spectra, for each of the 18 cases under study.

Cases	AUC ^1^ Ratio				AUC Ratio Reduction		
	pCCS	CCS	nCCS	Individual Spectra ^3^	pCCS vs. CCS ^2^	pCCS vs. nCCS	pCCS vs. Individual Spectra ^3^
D1	6.8%	8.9%	14.7%	12.7%	24.1%	54.1%	46.5%
D2	6.5%	8.7%	15.2%	13.0%	25.0%	57.3%	49.8%
D3	6.3%	8.2%	14.3%	12.7%	23.4%	56.0%	50.4%
D4	12.3%	17.1%	25.8%	19.4%	27.8%	52.3%	36.4%
D5	12.3%	17.0%	25.8%	19.4%	27.7%	52.5%	36.7%
D6	12.3%	16.8%	25.8%	20.2%	26.7%	52.2%	38.9%
D7	11.8%	16.7%	25.5%	19.0%	29.1%	53.6%	37.7%
D8	11.9%	16.7%	26.1%	19.6%	28.8%	54.5%	39.5%
D9	11.8%	16.4%	25.4%	19.4%	28.0%	53.4%	38.9%
D10	5.6%	7.8%	13.8%	11.6%	27.8%	59.4%	51.6%
D11	6.3%	8.7%	15.4%	12.0%	27.7%	59.2%	48.0%
D12	8.5%	11.4%	17.4%	14.5%	25.1%	51.1%	41.2%
D13	14.5%	19.6%	27.4%	20.3%	26.4%	47.3%	28.9%
D14	14.4%	19.5%	27.5%	20.4%	25.9%	47.6%	29.2%
D15	14.6%	19.5%	26.4%	20.1%	25.2%	44.7%	27.3%
D16	13.7%	18.9%	26.9%	20.5%	27.4%	49.1%	33.3%
D17	14.5%	19.5%	27.2%	20.6%	25.7%	46.6%	29.4%
D18	13.1%	18.2%	25.8%	19.8%	28.0%	49.3%	34.0%
Min.	5.6%	7.8%	13.8%	11.6%	23.4%	44.7%	27.3%
Max.	14.6%	19.6%	27.5%	20.6%	29.1%	59.4%	51.6%
Avg.	11.0%	15.0%	22.6%	17.5%	26.7%	52.2%	38.8%
Std. Dev.	3.3%	4.6%	5.5%	3.5%	1.6%	4.2%	7.8%

^1^ AUC (area under the curve) ratio: AUC of the 50% lower points/AUC of the total spectrum. ^2^ AUC ratio reduction: 1—pCCS AUC ratio/CCS AUC ratio. ^3^ Median of the four individual spectra.
